# Region-Specific Methylation Profiling in Acute Myeloid Leukemia

**DOI:** 10.1007/s12539-018-0285-4

**Published:** 2018-02-05

**Authors:** Agnieszka Cecotka, Joanna Polanska

**Affiliations:** Data Mining Division, Faculty of Automatic Control, Electronics and Computer Science, Institute of Automatic Control, Silesian University of Technology, ul. Akademicka 16, 44-100 Gliwice, Poland

**Keywords:** Epigenetics, DNA methylation, Differentially methylated regions, DMR, Data driven algorithm, Gaussian mixture modeling, Acute myeloid leukemia, AML

## Abstract

Alteration of DNA methylation level in cancer diseases leads to deregulation of gene expression—silencing of tumor suppressor genes and enhancing of protooncogenes. There are several tools devoted to the problem of identification of CpG sites' demethylation but majority of them focuses on single site level and does not allow for quantification of region methylation changes. The aim was to create an adaptive algorithm supporting detection of differentially methylated CpG sites and genomic regions specific for acute myeloid leukemia. Knowledge on AML methylation fingerprint helps in better understanding the epigenetics of leukemogenesis. Proposed algorithm is data driven and does not use predefined quantification thresholds. Gaussian mixture modeling supports classification of CpG sites to several levels of demethylation. *p* value integration allows for translation from single site demethylation to the demethylation of gene promoter and body regions. Methylation profiles of healthy controls and AML patients were examined (GEO:GSE63409). The differences in whole genome methylation profiles were observed. The methylation profile differs significantly among genomic regions. The lowest methylation level was observed for promoter regions, while sites from intergenic regions were by average higher methylated. The observed number of AML related down methylated sites has not substantially exceeded the expected number by chance. Intergenic regions were characterized by the highest percentage of AML up methylated sites. Methylation enhancement/diminution is the most frequent for intergenic region while methylation compensation (positive or negative) is specific for promoter regions. Functional analysis performed for AML down methylated or extreme high up methylated genes showed strong connection to the leukemic processes.

**Electronic supplementary material:**

The online version of this article (10.1007/s12539-018-0285-4) contains supplementary material, which is available to authorized users.

## Introduction

DNA methylation is one of gene expression regulatory mechanism, managed by epigenetic process of transformation cytosine into 5-methyl cytosine. This phenomenon occurs only in CpG sites, which is cytosine followed by guanine in a DNA strand [1]. The role of DNA methylation is best known for promoter (TSS) regions. Very high methylation level in this area leads to lock the initiation of transcription. There is also a hypothesis that high level of methylation in gene body region enhances elongation process, but it is still not confirmed [2]. In cancer diseases, hypermethylation on promoter regions of tumor suppressor genes leads to inhibition of their expression and hypomethylation on promoter regions of protooncogenes induces their higher expression [3].

The existing methylation data analysis methods base on parametric statistical tests for mean methylation levels [4]. The core of their functioning is detection of demethylated CpG sites across the genome only. Demethylated regions are defined by amount of demethylated CpG sites in examined region [5]. dmpFinder in minfi package for bioconductor is the most popular method for identification of demethylated sites [6]. More advanced algorithms consider the dependencies among CpG sites caused by their neighborhood [7].

The aim of the work is to develop a novel adaptive method of methylation data analysis that will lead to identification of demethylated both, single CpGs and regions of the genome. Proposed method is data driven and categorize methylation sites and genome regions as low, medium or high demethylated.

## Materials and Methods

The data set GSE63409 [8, 9] consists of raw methylation profiles measured by Infinium HumanMethylation450 microarray (Illumina) from five hematopoietic stem cells’ samples from healthy donors (HSC) and 14 samples of CD34 + 38-cells from AML patients. Each data file contains methylation level (defined as the fraction of methylated probes named as *β* value) for 485,512 CpG sites of human genome. *β* value ranges from 0 to 1, where 0 means no methylation and 1 means full methylation [10].

Data was normalized with R Bioconductor *minfi* package [6]. Following Illumina’s annotation system, each CpG site was assigned to its chromosome number, locus, probe sequence, RefGene Name and RefGene Accession (if present), RefGene Group, and Relation to CpG Island. Since the whole genome is divided into several regions according to the gene structure: intergenic, TSS1500, TSS200, 5′UTR, 1stExon, Body and 3′UTR regions, these classes were used to form RefGene Group’s options.

Kaplan–Meier estimate of empirical cumulative distribution function (ecdf) was computed for pooled samples [11]. Cohen’s *d* statistics was used to assess the effect size [12]. Verification of the hypothesis on consistency in methylation profiles was done by Cramer’s *V* coefficient [13]. The Hodges–Lehmann (*HL*) statistics was used to estimate the shift between the *β* value distributions of AML and healthy donors per each CpG site [14]. Its value denotes the level of demethylation. Significant positive value of *HL* statistics means site up methylation in AML patients, while negative is understood as site down methylation.

Gaussian mixture modeling (GMM) of *HL* distribution across genome was used to identify different demethylation levels. The expectation maximization (EM) algorithm for recursive maximization of the likelihood function was applied during the model fitting [15]. The initial values of GMM components were set according to the algorithm by Polanski et al. [16]. Bayesian information criterion (BIC) [17] was used for model selection. The data driven cutoff values were defined by maximum probability criterion and were equal to the intersection points of probability density functions of obtained Gaussian components.

Statistical testing was performed for each CpG site to detect significantly low or high methylated sites in both HSC and AML groups independently, and to identify up and down methylated sites in AML. Appropriate version of parametric *t* test or nonparametric one sample Wilcoxon or two sample Mann–Whitney tests were used to search for significantly demethylated sites (DMS) [18]. Results with *p* value less than 0.05 (in case of two-sided tests) or 0.025 (one-sided tests) were considered statistically significant at first step. In addition, using GMM based cut-off values, the hypotheses on relatively low, medium and high AML up or down methylation were verified. Storey’s [19] technique was used to correct for multiple testing.

Stouffer’s method [20] for *p* value integration was used to translate demethylation *p* values from CpG site to genome region level. The procedure was applied for each gene associated TSS and Body region. Functional analysis was performed by checking on overrepresentation of Gene Ontology [21, 22] terms for the identified set of demethylated genes. *TopGO* package for Bioconductor was used to perform overrepresentation analysis [23].

In addition, genome locations of CpG sites were examined for connection with long noncoding RNA, enhancers and transposable elements. Annotations for long noncoding RNA were downloaded from GENCODE project [24] webpage, for enhancers come from FANTOM5 project [25] resources, and annotations for transposable elements were found in UCSC Genome Browser [26]. Demethylated TSS regions where checked for being microRNA targets with miRWalk2.0 tool [27, 28].

## Results and Discussion

### Whole Genome Methylation Profile

**Fig. 1:**
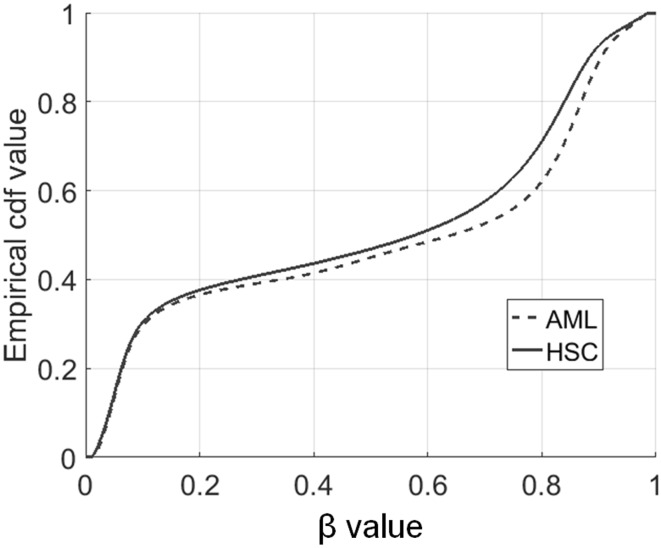
Whole genome pooled empirical cdf for HSC and AML samples

Pooled empirical cumulative distribution functions of whole genome CpG methylation level for both HSC and AML samples are presented in Fig. 1. The differences in whole genome methylation profiles between leukemia and healthy donors can be observed. The Cohen’s *d* statistics at the level of 0.2183 points out at small global effect size of AML on methylation profile. The HSC and AML distributions do not differ so much for small *β* values, but the distance between them increases for high methylation level (*β* > 0.5). The numbers for significantly low (*β* < 0.5) and high (*β* > 0.5) methylation sites in both groups presents Table 1, where CpG site was classified as low, medium, or high methylated depending on the results of Wilcoxon test with null hypothesis on *β* = 0.5.

**Table 1:** Number of low, medium, and high methylated CpG sites in HSC and AML samples

Methylation level	AML			
	Low	Medium	High	Total
HSC				
Low	191,043	14,739	2985	208,767
Medium	5668	11,286	33,931	50,885
High	2297	10,093	213,470	225,860
Total	199,008	36,118	250,386	485,512

The methylation profiles are in general consistent in both groups—majority of sites classified as low methylated in HSC were also classified as low methylated in AML (191,043 CpG sites), the same with high methylation status (213,470 CpG sites). Cramer’s *V* association coefficient for obtained contingency table was equal to 0.6667 (*p* value < 1e–6 [29]). The detailed inspection of Table 1 reveals, that there are more CpG sites that are high methylated in AML and low methylated in HSC compared to the opposite situation with low methylation in AML and high methylation in HSC. It suggests that more genes are up methylated than down methylated in AML. That hypothesis requires further investigation.

### Methylation Level in Different Genomic Region

Following information included in Infinium array annotation files, each site was assigned to one of three classes: transcription start site (TSS), gene Body, and Intergenic region. Sites with RefGene Group values: TSS1500, TSS200 or 5′UTR were included into TSS class, while sites annotated as: 1stExon, Body or 3′UTR constructed gene Body class. The sites do not annotated to any of these regions were considered as Intergenic. Due to gene overlapping, CpG site can be assigned to several RefGenes and/or several RefGene Groups. Table 2 presents the site counts for each class.

**Table 2:** Number of CpG sites assigned to each genome region

TSS region	Gene body region	Intergenic region
189,524	227,032	93,520

**Fig. 2:**
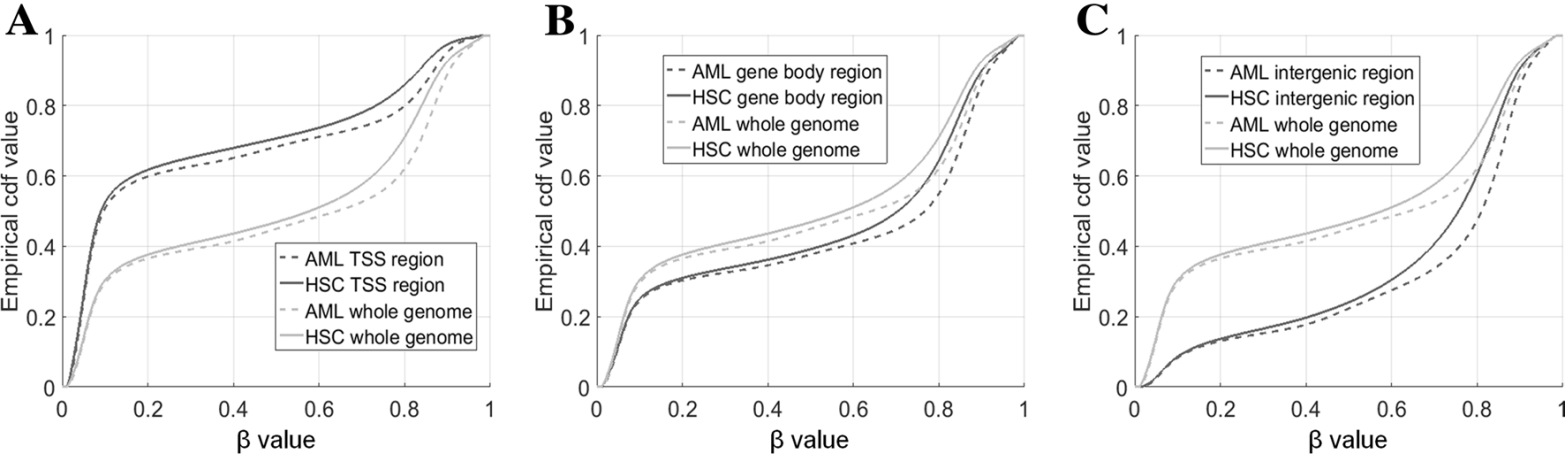
Empirical cdfs for **a** TSS, **b** gene body, and **c** intergenic regions

The pooled empirical cdf for each class in both, HSC and AML groups were estimated. Figure 2 presents the obtained curves. Methylation level for AMLs is slightly higher than for HSC in case of whole genome analysis and for region-specific separate analyses as well. The methylation profiles differ significantly among genomic regions. The lowest average methylation level is observed for sites from TSS regions, while sites from Intergenic regions are by average higher methylated. Methylation profile for gene Body sites does not differ so much from whole genome profile. That phenomenon is independent of disease status and is seen in both, HSC and AML samples. The association between significantly low/medium/high HSC and AML sites remains strong independently of genomic region.

If HSC are considered, more than 67% of CpG sites (128,145 of 189,524) located inside TSS region is low methylated, while only 19% of intergenic sites are from the same class (17,554 of 93,520). Similar trend is observed for AMLs—65% for TSS versus 16% for intergenic. The consistency of methylation profiles between HSC and AML data, as measured by Cramer’s *V*, keeps but is getting lower with stepping from TSS, gene body to intergenic regions (*V* equals to 0.6692, 0.6658, and 0.6119, respectively) (Table 3).

**Table 3:** Distribution of low, medium, and high methylated CpG sites in HSC and AML for different genomic regions

Methylation	AML											
	TSS region				Gene body region				Intergenic region			
	Low	Medium	High	Total	Low	Medium	High	Total	Low	Medium	High	Total
HSC												
Low	121,393	5693	1059	**128,145**	73,494	5874	1300	**80,668**	13,548	3295	711	**17,554**
Medium	2101	3578	8643	**14,322**	2417	4639	16,438	**23,494**	1154	3065	9893	**14,112**
High	614	2741	43,702	**47,057**	1087	4661	117,122	**122,870**	559	2722	58,573	**61,854**
Total	**124,108**	**12,012**	**53,404**	**189,524**	**76,998**	**15,174**	**134,860**	**227,032**	**15,261**	**9,082**	**69,177**	**93,520**

### Methylation Signature of AML

Standard approach in searching for differentially demethylated sites calls for statistical testing of the hypothesis on no mean/median difference in *β* methylation level between two populations (HSC and AML in our case). The results of such approach are presented in Table 4.

**Table 4:** Total and region-specific number of differentially demethylated sites—unadjusted *p* values (one-side tests, significance level *α* = 0.025)

	Whole genome		TSS region		Gene body		Intergenic region	
	Down	Up	Down	Up	Down	Up	Down	Up
Significantly AML demethylated sites	15,260 (3.14%)	84,073 (17.32%)	5287 (2.79%)	28,492 (15.03%)	7010 (3.09%)	39,622 (17.45%)	3075 (3.29%)	19,737 (21.10%)

The false discovery rate (FDR) for identification of AML down methylated sites is high independently on genomic region. The observed number of significantly down methylated CpG sites does not substantially exceed the expected number by chance (2.50%). The lowest fraction was detected for sites from TSS regions (2.79%), while the highest fraction of CpG sites distinguishing AML from HSC was revealed for intergenic region. *FDR* value decreases drastically when the up methylation is considered. As for the down methylation, the highest fraction of significantly up methylated sites was observed for intergenic region (21.10%) with rough *FDR* estimate equal to 8.14%. The number of TSS region-specific AML up methylated sites was slightly less (15.03%) but still *FDR* stays at acceptable level.

**Fig. 3:**
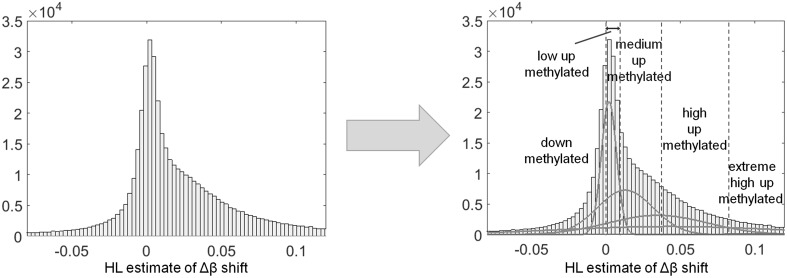
Distribution of HL statistics, its GM model, and the identified classes of low, medium, high and extreme high AML up methylation

Knowing that there are CpG sites significantly up methylated in AML, it is inviting to classify them as low, medium or high up methylated. To obtain the data driven cut-off values, a novel approach was developed. Hodges-Lehmann (*HL*) estimate, representing a shift between AML and HSC *β* level distributions, was calculated for each CpG site and the obtained distribution of *HL* values was modelled as a mixture of Gaussian components. Figure 3 presents both, *HL* distribution and its GM model, while Table 5 gives the parameters of mixture components—their mean value, standard deviation and mixture fraction (weight).

**Table 5:** Parameters of the HL related GMM components

Component ID	Mean value	Standard deviation	Weight	Component ID	Mean value	Standard deviation	Weight
1	0.0128	0.0189	0.2645	5	0.1792	0.1269	0.0597
2	0.0019	0.0051	0.2148	6	− 0.1248	0.1107	0.0358
3	0.0348	0.0334	0.2045	7	− 0.3006	0.1940	0.0158
4	0.0427	0.0748	0.1942	9	0.3818	0.1775	0.0107

First four components describe 87.80% of signal in total, and are of similar weight, but their dispersion increases with increasing Δ*β* shift (estimated by component location statistics—mean value). The remaining part is modelled by additional four components of significantly lower weights. Since the *HL* distribution is right skewed (skewness *γ*_1_ = 0.1408) Gaussian component located close to 0 value (no difference between HSC and AML) is accompanied by three additional components, centered at Δ*β* equal to 0.01, 0.03, and 0.04, respectively.

The maximum probability criterion [30] allowed to construct a set of conditions needed for classification of site up methylation level. CpG site with statistically significant Δ*β* = *β*_AML_ − *β*_HSC_ > 0 are classified as “AML up methylated”, with Δ*β* > 0.0096 as “at least medium up methylated”, those with significant Δ*β* > 0.0372 as “at least high up methylated” and those with Δ*β* > 0.0819 as “extreme high up methylated”. Table 6 presents the results of site classification depending on the strength of AML versus HSC up methylation.

**Table 6:** Number of significantly AML up methylated CpG sites depending on genomic region and up methylation level

Level of AML demethylation	Whole genome		TSS region		Gene body		Intergenic	
	*N*	%	*N*	%	*N*	%	*N*	%
Up methylation	84,073	17.32	28,492	15.03	39,622	17.45	19,737	21.10
At least medium	47,659	9.82	14,196	7.49	22,177	9.77	12,738	13.62
At least high	17,317	3.57	5577	2.94	7414	3.27	4734	5.06
Extreme high	8149	1.86	2716	1.43	3477	1.53	2142	2.29

Intergenic region is characterized by the highest percentage of AML up methylated CpG sites independently of the up methylation level. For at least medium AML up methylation, its occurrence is 1.8 times more frequent than in TSS region. In case of at least high AML up methylation the ratio between number of events within intergenic and TSS is very similar and it is equal to 1.7. While extreme high up methylation is considered, the difference between genomic regions vanishes and the number of differentially methylated sites gets closer to the expected by chance.

### Dynamics of AML Specific Demethylation Processes

Complete knowledge on AML related demethylation profile requires investigation on the relation of that process to the initial level of CpG methylation in healthy donors. Table 7 presents the connection between HSC low/medium/high site methylation status and results of AML versus HSC comparison study.

**Table 7:** AML up and down methylation in relation to HSC methylation status

	AML demethylation	HSC low	HSC medium	HSC high
		*N*	*N*	*N*
Whole genome	Down	5374	2373	7513
	No change	172,711	37,773	175,735
	Up	30,682	10,779	42,612
TSS	Down	2764	774	1749
	No change	109,047	10,694	36,014
	Up	16,334	2864	9294
Body	Down	2189	1046	3775
	No change	66,433	17,439	96,528
	Up	12,046	5009	22,567
Intergenic	Down	523	535	2017
	No change	12,575	10,364	47,769
	Up	4456	3213	12,068

While the whole genome analysis is performed, third part of CpG sites are of HSC low and AML unchanged type (35.57%). The similar percentage (36.19%) is specific for HSC high and AML unchanged. Next the most frequent response is AML up methylation of HSC high methylated sites (8.78%). Frequencies of particular classes differ among genomic regions. The alterations in DNA methylation of TSS region varies from similar processes in body and intergenic regions. The most numerous class is defined as HSC low methylation and no impact of AML (57.33% of sites), whilst is much less frequent in body (29.26%) and intergenic regions (13.45% of sites). Whereas methylation dynamics in response to AML is investigated two major types can be defined: (1) *methylation enhancement*/*diminution* with two situations possible—HSC low methylated site gets lower by down methylation in AML or HSC high methylated site is additionally up methylated in AML and (2) *methylation compensation*, where reverse process is observed—HSC low methylated site gets up methylated in AML or HSC high methylated gets down methylated in AML. These two reactions are almost balanced in whole genome analysis (9.88% in methylation enhancement/diminution versus 7.87% for methylation compensation), but they look different when separate regions are considered. In case of methylation enhancement/diminution, the frequency equals to 6.36% for TSS sites, rises to 10.90% for gene Body located sites to almost double for intergenic region (13.46%). The reverse AML response process, named methylation compensation, is of similar frequency in Body and Intergenic regions (6.97 and 6.92%, respectively) and 1.5 times increases for TSS region (9.54% if these sites).

AML up methylated sites located in TSS are primary low methylated in HSC. That finding is on the contrary to up methylation process within gene body and intergenic regions, where more up methylated sites is originally of high methylation level in HSC (56.96% for gene body region and 61.14% for intergenic region). From the other side, if only HSC low methylation sites are considered within each region independently, 25.38% of such sites in intergenic gets up methylated, and only 12.75% of TSS located sites. HSC high methylated sites get up methylated by AML at similar level, independently of genomic region (19.75% for TSS, 18.37% for Body, and 19.51% for Intergenic). Since regions of high density of CpG sites (recognized as CpG island) are located within TSS regions, our results suggest that the most of modifications in DNA methylation caused by AML are probably inside CpG islands.

### From Demethylated Sites to Demethylated Genes

Translation from single site to gene level was done based on genomic CpG location and RefGene Name and RefGene Group values. The information on demethylation of all sites assigned to the TSS region of same gene was integrated by Stouffer method giving significance of up or down methylation of TSS region. Similar operation was done for gene Body regions. Infinium HumanMethylation450 microarray covers 21,227 genes in total by having at least one site located in their TSS (20,852 genes) or Body region (20,527 genes). Table 8 presents the summary of results integration. The complete list of demethylated genes is given in Supplementary materials #1 and #2.

**Table 8:** Number of demethylated genes after *p* value integration with respect to demethylated TSS and gene body regions

AML associated demethylation at gene level	Unadjusted *p* values				Storey’s corrected *p* values			
	Genes with demethylated TSS region		Genes with demethylated Body region		Genes with demethylated TSS region		Genes with demethylated Body region	
	*N*	%	*N*	%	*N*	%	*N*	%
Down methylation	90	0.43	112	0.55	22	0.11	14	0.07
Up methylation	945	4.53	948	4.62	600	2.88	598	2.91
At least medium	385	1.85	422	2.06	187	0.90	162	0.79
At least high	105	0.50	115	0.56	53	0.25	25	0.12
Extreme high	31	0.15	35	0.17	18	0.09	5	0.02

Among genes with extreme high up methylation of TSS are: *SCG5, OXT, CRHBP, WDR52, RHD, MFSD6L, PCDHGA6, CMYA5, KRTCAP3, CCDC81, SIAH3, CYP26C1, LOC254559, HTRA4, ACOX2, SPACA1, RSPH10B* and *RSPH10B2*, while the list of genes with down methylated TSS region includes among the other genes: *PRF1, TRPM2, LOC150381, CCL3, IL10, CXCR3, CHRNA6, ESPNL, CFD, C17orf87, KRT17, GPR62, CD68, MIR320C1, LILRB3, CD19, PRDM11, CCL22, LOC387647, NKG7, TYR* and *ITGAX*. If gene body is considered, the number of demethylated genes decreases, among extreme up methylated are: *ELTD1, HTRA4, UCN, TMPRSS12* and *C6orf146*. Genes with down methylation in body region are: *C22orf26, FUT4, NCF4, C1orf129, LCE3A, HCST, DNAJB5, OR9G1, OR9G9, OR6M1, C6orf164, GRAP2, OR8H3*, and *RNASE3*.

**Fig. 4:**
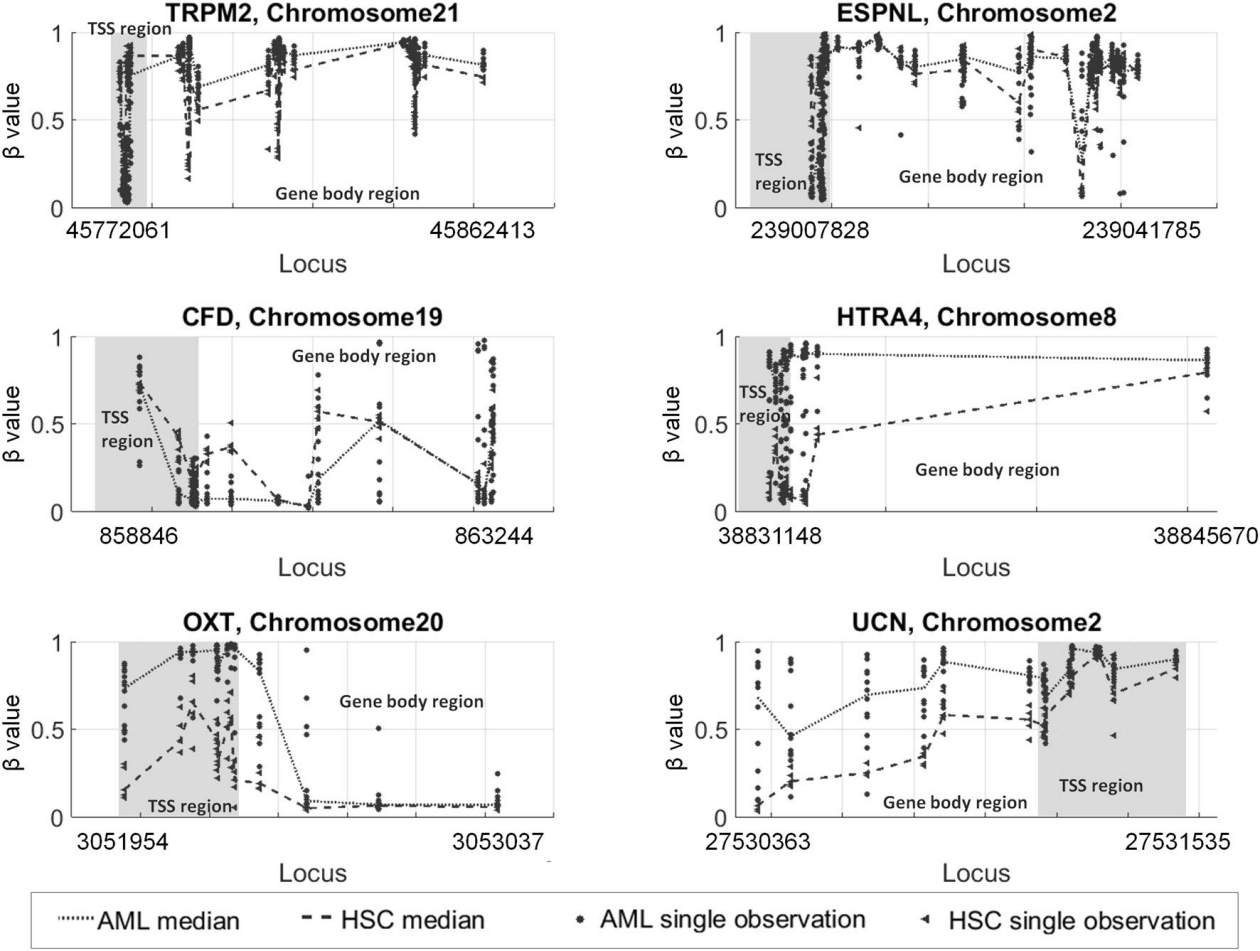
Methylation profiles for exemplary genes

*TRPM2* gene TSS region is down-methylated in AML (Fig. 4). Higher expression of *TRPM2* was observed in several tumor family diseases such as insulinoma, hepatocellular carcinoma, prostate cancer, lymphoma, leukemia, and lung cancer cell lines. In these cases *TRPM2* could enhance cell death [31]. *ESPNL* gene shows hypomethylation in MDS (Myelodysplastic syndrome), which is often precursor of AML. *ESPNL* gene is considered crucial in epigenetic drift related to age in the pathogenesis of MDS and AML [32]. Down methylated gene, *CFD*, is main regulator of complement activation and may advantage leukemia aggressiveness by suppression of the immune response to AML and regulation of stem cell function [33]. *HTRA4* was detect as extremely high up methylated in TSS as well as in gene body regions. It is confirmed to be tumor suppressor gene and consider as biomarker in other cancers [34]. It was also described as down regulated in AML. Extremely high up methylated *OXT* gene characterizes decreased activity in Chronic Myeloid Leukemia, in comparison to healthy control. In addition, it has lower activity and expression in CD34+ cell (which were used in presented study) than in CD34− [35]. *UCN* gene, coding protein kinase C inhibitor, was detected as extreme high up methylated in gene body region. It was described as apoptosis inducing in Human and Leukemia Cells Independently of *p53* in treatment of human myeloblastic leukemia [36]. *MYOD1* found hypermethylated in AML [37] was detected by us as up methylated in TSS region. *CDH1* and *HIC1*, mentioned as AML high methylated in [38] were detected as up methylated in TSS region (*CDH1)* and up methylated in gene body region (*HIC1*). *DPP6* and *ID4* identified as AML up methylated in promoter region and with their expression being down regulated [39] were detected in our study as medium up methylated in TSS region.

### Functional Analysis of Down and Up Methylated Genes

Gene Ontology based functional analysis was performed on TSS down methylated, TSS extreme high up methylated, Body down methylated and TSS extreme high up methylated gene sets separately. Summary of GO functional analysis is presented in Table 9. The complete information on significantly overrepresented GO terms in given in Supplementary materials #3 and #4.

**Table 9:** Number of significantly overrepresented GO terms

Gene Ontology terms	TSS down	TSS extreme high	Body down	Body extreme high
Biological process	113	74	8	56
Molecular function	13	4	7	2
Cellular component	25	7	10	0

A lot of GO terms detected for TSS down methylated genes were connected to calcium ion transport and sequestering (for example: *GO:0051283, GO:0051282, GO:0060402, GO:0070588, GO:0060401, GO:0010857, GO:0009931*) which is consistent with literature findings where the alteration in calcium processes in AML is very commonly reported [40]. The second group of GO terms detected for TSS down methylated genes are processes concerning immune system, which is concordant with AML as an immune system disease. Examples of these processes are: leukocyte differentiation (*GO:0002521*), hematopoietic or lymphoid organ development (*GO:0048534*), regulation of interleukin-1 production (*GO:0032652*), negative regulation of myeloid cell differentiation (*GO:0045638*), regulation of cytokine secretion (*GO:0050707*) and many more [41].

Some GO Terms overrepresented in TSS extreme high up methylated genes are connected to hormone metabolic processes, especially estrogen (*GO:0042445, GO:0032355, GO:0071391, GO:0010817, GO:0046883, GO:0009914, GO:0042562*). Estrogen receptor gene was described as cancer biomarker and despite it is not highly demethylated in our study, processes directly connected to it were detected [42, 43]. Some overrepresented GO Terms for the same gene group concern response for drugs and steroids, ex. alkaloids, alcohol, cocaine (*GO:0042220, GO:0008202, GO:0097305, GO:0045472, GO:0043279*). Affective of drugs is usually bigger in tumors [44].

Most of GO Terms detected for gene body regions could be found by chance. Only for extreme high up methylated genes in body region are some interesting results. Big part of them are connected to collagen processes (*GO:0032964, GO:0010712, GO:0032965, GO:0010714, GO:0032967*).

### Investigation for Long Noncoding RNAs, Enhancers, Transposable Elements and microRNAs

Annotation file with genome location of long noncoding RNAs contains 51,893 lincRNAs. In 13,266 of them, at least one CpG site was found. While more than one CpG site was found across one lincRNA, *p* value of them were integrated, analogously like in TSS or Body genome regions. Number of demethylated lincRNAs is presented in Table 10. Analysis for enhancers and transposable elements was performed in the similar way. 1827 of 32,216 enhancers contains at least one CpG site, while 29,174 of 575,600 transposable elements contains at least one CpG site. Number of demethylated enhancers and transposable elements is also presented in Table 10. The comprehensive lists are presented in Supplementary material #5.

**Table 10:** Number of demethylated lincRNAs, enhancers and transposable elements

AML demethylation	Down methylation	Up methylation	At least medium	At least high	Extreme high
linc RNAs	289	1368	814	269	112
Enhancers	74	262	143	53	19
Transposable elements	838	5325	3111	727	180

Investigation for microRNA targets was performed for sets of demethylated TSS regions: TSS down methylated and TSS at least high up methylated. Analysis of TSS extremely high up methylated regions did not give any results. TSS down methylated regions are targets for 271 microRNAs and TSS at least high up methylated regions are targets for 222 microRNAs. The details can be found in Supplementary materials #5.

### Comparison to dmpFinder Based Results

*dmpFinder* is a commonly used algorithm implemented in R Bioconductor (*minfi*) package. We compared our findings to *dmpFinder* results. Our nonparametric method detects 99,333 CpG sites as demethylated while dmpFinder detects 97,596 CpG sites. 71,244 of them are the same CpG sites. After *p* value correction, dmpFinder detects 29,609 CpG sites and our algorithms identifies 28,089 demethylated CpG sites, 18,367 of them were the same as from *dmpFinder*. Dice index [45] is equal to 64%.

## Conclusions

Novel method for methylation data analysis was proposed allowing for not only efficient detection of demethylated CpG sites but also demethylated genes and genomic regions. AML genome wide methylation fingerprint was identified with the use of developed technique. The algorithm uses well attuned statistical methods supported by mathematical modeling. In contrary to existing approaches, it is data driven and does not use a priori assumed cutoffs for demethylation definition. Thanks to Gaussian mixture modelling of distribution of methylation shift between groups, it allows to classify CpG sites as low, medium or high demethylated with the support of probability for class membership. Due to *p* value integration our approach enables to conclude about demethylation of particular TSS and gene body regions.

Study confirmed that acute myeloid leukemia causes alterations in DNA methylation. The AML methylation modification is different for different genomic regions: TSS, gene body and intergenic. Much more CpG sites and regions were detected as up methylated than down methylated. Low and high methylated sites changes more than medium methylated. AML caused down and up methylated genes found, especially with significant modifications in TSS region, were confirmed as directly connected to leukemia. Functional analysis shows relationship between found genes and processes alternated in AML.

## Electronic supplementary material

Supplementary material 1 (XLSX 3588 KB)

Supplementary material 2 (XLSX 2558 KB)

Supplementary material 3 (XLSX 28 KB)

Supplementary material 4 (XLSX 17 KB)

Supplementary material 5 (XLSX 3988 KB)

## Abbreviations

DNA: Deoxyribonucleic acid
TSS: Transcription start size
GMM: Gaussian mixture modeling
AML: Acute myeloid leukemia
Cdf: Cumulative distribution function
